# A relationship between species richness and evenness that depends on specific relative abundance distribution

**DOI:** 10.7717/peerj.4951

**Published:** 2018-06-12

**Authors:** Qiang Su

**Affiliations:** College of Earth and Planetary Sciences, University of Chinese Academy of Sciences, Beijing, China

**Keywords:** Index, Diversity, Fractal theory, Species abundance distribution

## Abstract

Although many ecologists focus on the relationship between species richness (*S*) and evenness (*E*), conflicts between observation and theory are difficult to reconcile. Empirical *S*–*E* relationships were not consistent, while relationships show strong correlation between *S* and *E*. Since *E* essentially depended on the relative abundance distribution (RAD), the hypothesis of this paper was that the *S*–*E* relationship should be determined by RAD. Theoretical *S*–*E* relationships for various RADs have already been reported, but they were rarely assessed by the raw data. This study constructed *S*–*E* relationships for a specific RAD, which indicated that if the community had a fractal distribution of rank abundance, *E* would decrease with *S*, and the *S*–*E* relationship would be unique for a given RAD. Such theoretical expectations were supported by three datasets with 82 samples, which suggested that the *S*–*E* relationship were controlled by RAD and inconsistent* S*–*E* relationships in statistical analyses could be accounted for by the variation of underlying RAD model between communities. From the perspective of RAD, it could be too early to split the diversity into *S* and *E* only based on the *S*–*E* relationship in statistical analyses.

## Introduction

The relationship between species richness (*S*, the number of species in a community) and evenness (*E*, the equitability of the proportional abundances of species) remains an unsettled issue in current ecology (Tuomisto, 2012). Many ecologists focus on the *S*–*E* relationship as it relates to an essential topic that the diversity is (or is not) a compound quantity made up of *S* and *E* (Jost, 2010). An independent *S*–*E* relationship suggests that the diversity can be split (Stirling & Wilsey, 2001; Wilsey & Stirling, 2007). Although many authors tend to treat *S* and *E* as independent (Stirling & Wilsey, 2001; Jost, 2010; Tuomisto, 2012) and hold an implicit view that *E* must be unaffected by *S* (Smith & Wilson, 1996), the *S*–*E* relationship seemed to be highly conflicting between theoretical and empirical perspectives (Zhang et al., 2012).

On one hand, Stirling & Wilsey (2001) found no consistent *S*–*E* relationships across different taxonomic categories by analyzing comprehensive datasets. Inconsistent *S*–*E* relationships were also present in many investigations, including human-shaped ecosystem (Ma, 2005), developing microcosm (Wilsey & Stirling, 2007), birds across Australia (Symonds & Johnson, 2008) and aquatic ecosystem (Soininen, Passy & Hillebrand, 2012). Some studies, including plant communities of plateau ecosystem (Zhang et al., 2012), grassland of America (Wilsey et al., 2005) and the neotropical bat community (Estrada-Villegas, McGill & Kalko, 2012), suggested that *S*–*E* relationships could be negative. In short, there is little consensus on empirical *S*–*E* relationships (Symonds & Johnson, 2008).

On the other hand, theoretical analyses suggested that *S*–*E* relationships were correlated (DeBenedictis, 1973; Gosselin, 2006). DeBenedictis (1973) stated that *S* was strongly positive with *E*. Hill (1973) hypothesized that *S* and *E* were related. Smith & Wilson (1996) tested fourteen evenness indices and found that several indices failed the requirement of independence of *S*. Jost (2010) and Tuomisto (2012) also suggested that *E* was actually constrained by *S*. Thus, *E* was affected by *S* in many theoretical cases.

In brief, empirical *S*–*E* relationships were not consistent (Stirling & Wilsey, 2001; Wilsey & Stirling, 2007; Zhang et al., 2012), and their theoretical relationships were correlated (DeBenedictis, 1973; Hill, 1973; Jost, 2010; Tuomisto, 2012). If this is so, does species diversity consist of *S* and *E*? Is there a general understanding that can reconcile the conflict between theory and observation?

In fact, *E* essentially relies on the relative abundance distribution (RAD) (Gosselin, 2006), which is a description of the proportional abundance for each species in a community (May, 1975; Tokeshi, 1993; McGill et al., 2007). Krebs (1989) stated that *E* was basically determined by RAD. The dependence of *E* on *S* is observed to be strong for most RADs, although they were rarely assessed by the raw data (Gosselin, 2006). Thus, *S*–*E* relationships could be controlled by RAD.

Altogether, this paper continued the study of evenness indices variation with species richness (Gosselin, 2006), but with a specific RAD (Su, 2016). Since there is only one parameter in this model, the empirical *S*–*E* relationship for each community can be indicated on *S*–*E* plots, which is the originality of this paper. The hypothesis of this paper is that the *S*–*E* relationship relies on the pattern of RAD, which thereby determines the controversial issue that the diversity is (or is not) a compound quantity made up of *S* and *E*. Objectives are to (1) construct the *S*–*E* relationship for a specific RAD (methods and results); (2) assess such relationships using raw data (results); (3) clarify the dependence of *S*–*E* relationships on this RAD model (results and discussion); (4) discuss the controversial issue that the diversity is (or is not) a compound quantity.

## Methods

Firstly, many different models have been proposed as descriptions of RAD (Baldridge et al., 2016), and which model gave the best fit to the raw data was unknown (Magurran, 1988; McGill et al., 2007; Baldridge et al., 2016). In this paper, a new fractal model of RAD (Su, 2016) is selected for two reasons: (1) Its hypothesis is easy to fit (McGill et al., 2007); (2) The *S*–*E* relationship for this fractal model has not yet been figured out (May, 1975; Gosselin, 2006; Su, 2016).

According to the fractal hypothesis (when *K* more species appear at each step of the accumulation process, their abundance are *k* times less abundant and *K* = *k*^*d*^, where *d* is a fractal dimension (Mouillot et al., 2000)), RAD can be described as follows (Su, 2016),

(1) \begin{eqnarray*}& & \frac{{A}_{r}}{{A}_{1}} ={r}^{-p}\end{eqnarray*}

where *r* (=1, 2, 3, … *S*) is the rank of species sorted down by species abundance; *A*_1_ and *A*_*r*_ are the abundance of dominant and the *r*-th species; *p* (=1∕*d*) is the fractal parameter and *p* > 0 (Su, 2016). Let *F*_*r*_ = ln(*A*_*r*_/*A*_1_) and *D*_*r*_ = ln(*r*). By minimizing the sum of squared error (that is ${\mathop{\sum }\nolimits }_{r=1}^{S}(-p{D}_{r}-{F}_{r})^{2}$) (Su, 2016), the fractal *p* is

(2) \begin{eqnarray*}& & p= \frac{-\sum _{r=1}^{S}{D}_{r}{F}_{r}}{\sum _{r=1}^{S}{D}_{r}^{2}} .\end{eqnarray*}

*p* determines the pattern of the RAD. Lower *p* means a slower decrease in *A*_*r*_ compared with *A*_1_ (higher evenness), and higher *p* indicates a rapid decrease (lower evenness). For a given *p*, the *S*–*E* relationship will be unique (Su, 2016).

Secondly, there are also many different ways to quantify *E* in a community (Tuomisto, 2012). Gosselin (2006) suggested that *S*–*E* relationships for some evenness indices were very similar. This paper actually continued the study of evenness indices variation with species richness (Gosselin, 2006), but with a specific RAD (Su, 2016). For the sake of comparison, four indices were selected, including Pielou’s evenness index (*E*_Pielou_) (Magurran, 1988), Hill’s evenness index (*E*_Hill_) (Hill, 1973), Bulla’s evenness index (*E*_Bulla_) (Smith & Wilson, 1996) and *E*_var_ (Smith & Wilson, 1996; Gosselin, 2006).

The total abundance is *A*_*T*_ = ∑*A*_*r*_. The relative abundance of the *r*-th species is

(3) \begin{eqnarray*}& & {p}_{r}= \frac{{A}_{r}}{{A}_{T}} .\end{eqnarray*}

Four evenness indices are

\begin{eqnarray*}& {E}_{\mathrm{Pielou}}= \frac{-\sum _{r=1}^{S}{p}_{r}\mathrm{ln}({p}_{r})}{\mathrm{ln}(S)} \end{eqnarray*}

\begin{eqnarray*}& {E}_{\mathrm{Hill}}= \frac{1/\sum _{r=1}^{S}{p}_{r}^{2}}{\mathrm{Exp} \left[ -\sum _{r=1}^{S}{p}_{r}\mathrm{ln}({p}_{r}) \right] } \end{eqnarray*}

\begin{eqnarray*}& {E}_{\mathrm{Bulla}}= \frac{ \left[ \sum _{r=1}^{S}\mathrm{min}({p}_{r},1/S) \right] -1/S}{1-1/S} \end{eqnarray*}

\begin{eqnarray*}& {E}_{\mathrm{var}}=1- \frac{2}{\pi } \mathrm{arctan} \left( \frac{\sum _{r=1}^{S}{ \left\{ \mathrm{ln}({p}_{r})-\sum _{i=1}^{S}[\mathrm{ln}({p}_{i})/S] \right\} }^{2}}{S} \right) . \end{eqnarray*}

Finally, Eq. (3) can be rewritten according to Eq. (1).

(4) \begin{eqnarray*}& & {p}_{r}= \frac{{A}_{r}}{{A}_{T}} = \frac{{A}_{1}}{{A}_{T}} \cdot \frac{{A}_{r}}{{A}_{1}} = \frac{{A}_{1}}{{A}_{T}} \cdot {r}^{-p}.\end{eqnarray*}

The *S*–*E* relationship for this fractal RAD can be achieved by entering Eq. (4) into four evenness indices.

How are the empirical data sets analyzed? If species abundance distribution of a community (form high to low) is as follows,

24, 12, 8, 6,

RAD of this sample (*A*_*r*_/*A*_1_, see Eq. (1)) will be

1, 1/2, 1/3, 1/4.

The fractal *p* and four evenness indices can be calculated by Eq. (2) and four indices equations. Then, *S*–*E* relationships can be colored in the scatter plot as the fractal *p* and evenness indices are known.

How is the *S*–*E* relationship for a specific *p* obtained? For example, when *p* = 1 and *S* = 6, RAD (*A*_*r*_/*A*_1_) should be

1, 1/2, 1/3, 1/4, 1/5, 1/6

when *p* = 1 and *S* = 7, RAD (*A*_*r*_/*A*_1_) should be

1, 1/2, 1/3, 1/4, 1/5, 1/6, 1/7.

Thus, when *S* and *p* are known, RAD and evenness indices should be given. With increasing *S*, the *S*–*E* relationship for a specific *p* (e.g., *p* = 1) can be obtained. In this paper, four *S*–*E* relationships (*S*–*E*_Pielou_, *S*–*E*_Hill_, *S*–*E*_Bulla_ and *S*–*E*_var_) were shown when *p* = 0.6, 1.2, 1.8 and 2.4.

### Datasets

Three datasets with totally 82 samples, including birds on island near Finland (Haila, 1983), stream fishes in the Otter Creek drainage basin (Harrel, Davis & Dorris, 1967) and zooplankton in the Tsugaru-Juniko lakes (Akifumi Ohtaka & Shoichi, 1996), are used for three reasons: (1) these samples are under different environments and have broad representations; (2) empirical *S*–*E* relationships for these data are unclear; (3) published datasets are easy to recheck.

## Results

**Figure 1:**
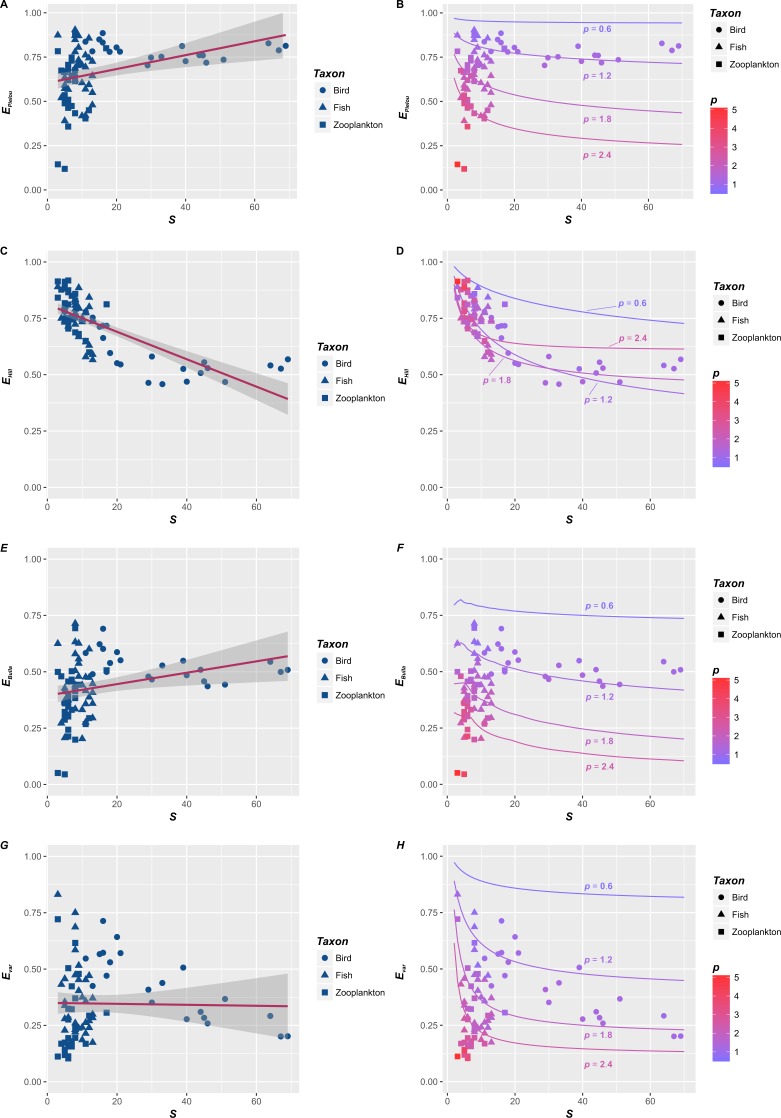
The relationships between species richness (*S*) and four evenness indices (*E*_Pielou_, *E*_Hill_, *E*_Bulla_ and *E*_var_) in three taxonomic categories. The relationships between species richness (*S*) and four evenness indices (*E*_Pielou_, *E*_Hill_, *E*_Bulla_ and *E*_var_) in three taxonomic categories, including birds (*circle*, Haila, 1983), fishes (*triangle*, Harrel, Davis & Dorris, 1967) and zooplankton (*square*, Akifumi Ohtaka & Shoichi, 1996). Basing on the ordinary linear regression, the *S*–*E* relationships were inconsistent (A, C, E and F). They could be positive (e.g., A and E), negative (e.g., C) or unrelated (e.g., F). However, reevaluating their relationships (points in B, D, F and H) suggested that they were essentially determined by *p*, which were consist with the theoretical *S*–*E* relationships corresponding to the fractal distribution with *p* = 0.6, 1.2, 1.8 and 2.4 (lines in B, D, F and H). In here, *p* is the fractal parameter. Lower *p* (cold color) means a slower decrease in *A*_*r*_ (the abundance of *r*-th species) compared with *A*_1_ (the abundance of dominant), and higher *p* (warm color) indicates a rapid decrease, where *r* is the rank of species sorted down by species abundance.

Firstly, empirical *S*–*E* relationships for four indices are not consistent (Figs. 1A, 1C, 1E and 1G). Basing on the ordinary linear regression, the *S*–*E*_Pielou_ relationship for the entire dataset (Fig. 1A) is weakly positive (*R*^2^ =0.125). *S*–*E*_Bulla_ and *S*–*E*_var_ relationships are both insignificant, and their *R*^2^ are 0.0775 and 0.0004, respectively. The negative *S*–*E*_Hill_ relationship (Fig. 1C) is relatively strong (*R*^2^ =0.543). *S*–*E* relationships also vary among taxonomic categories. Separated figures of *S*–*E* relationships for three communities within specific intervals of *p* values were provide in supplementary material. *S*–*E* relationships for four indices are all negative in bird (Haila, 1983) and fish (Harrel, Davis & Dorris, 1967) (Table 1). However, *S*–*E* relationships of zooplankton (Akifumi Ohtaka & Shoichi, 1996) are positive except the *S*–*E*_Hill_ relationship (Table 1). In brief, empirical *S*–*E* relationships for four indices are not consistent across three taxonomic categories (Table 1).

**Table 1:** The simple linear relationships between species richness (*S*) and four evenness indices (*E*_Pielou_, *E*_Hill_, *E*_Bulla_ and *E*_var_) in three taxonomic categories, including birds (Haila, 1983), fishes (Harrel, Davis & Dorris, 1967) and zooplankton (Akifumi Ohtaka & Shoichi, 1996). The *S*–*E* relationships for four indices are all negative in bird and fish, but in zooplankton, they are positive (except *E*_Hill_). *n* is the number of community samples. The maximum value of *R*^2^ is 0.6967 (the *S*–*E*_var_ relationship in birds) and 0.5430 (the *S*–*E*_Hill_ relationship for the entire dataset). The rest of *R*^2^ are all lower than 0.4, which indicates that the empirical *S*–*E* relationships for four indices are not very strong.

Communities	Bird (*n* = 21)	Fish (*n* = 33)	Zooplankton (*n* = 28)	Total data (*n* = 82)
*E*_Pielou_ vs *S*	*y* = − 0.0007*x* + 0.809	*y* = − 0.0006*x* + 0.674	*y* = 0.0172*x* + 0.440	*y* = 0.0172*x* + 0.440
*R*^2^/SE^a^	0.0766/0.0462	0.0002/0.1423	0.0738/0.1766	0.0738/0.1541
*E*_Hill_ vs *S*	*y* = − 0.0034*x* + 0.697	*y* = − 0.0119*x* + 0.858	*y* = − 0.0148*x* + 0.898	*y* = − 0.0148*x* + 0.898
*R*^2^/SE^a^	0.3968/0.0804	0.1837/0.0738	0.2431/0.0757	0.2431/0.0828
*E*_Bullar_ vs *S*	*y* = − 0.0017*x* + 0.584	*y* = − 0.0014*x* + 0.448	*y* = 0.0124*x* + 0.270	*y* = 0.0124*x* + 0.270
*R*^2^/SE^a^	0.2431/0.0570	0.0011/0.1269	0.0649/0.1358	0.0649/0.1293
*E*_var_ vs *S*	*y* = − 0.0067*x* + 0.649	*y* = − 0.0193*x* + 0.541	*y* = 0.007*x* + 0.213	*y* = 0.007*x* + 0.213
*R*^2^/SE^a^	0.6967/0.0835	0.1181/0.1549	0.0153/0.1626	0.0153/0.1708

**Notes.**

a The confidence intervals for the regression analyses is 95%.

Secondly, when *p* is fixed, *S*–*E* relationships for the fractal RAD (Su, 2016) are negative (lines in Figs. 1B, 1D, 1F and 1H). Generally, four evenness indices all decline with *S*, which is consistent with previous theoretical relationships for various RADs (Gosselin, 2006). Specifically, evenness indices (*E*_Pielou_, *E*_Bulla_ and *E*_var_) decline faster with *S* when *p* is larger (red lines in Figs. 1B, 1F and 1H). However, the impact of *p* on the *S*–*E*_Hill_ relationship is slightly complex (Fig. 1D). Although the *S*–*E*_Hill_ relationship is overall unique for a given *p* (Fig. 1D), decreasing *E*_Hill_ with *S* in the case of lower *p* (blue lines in Fig. 1D) probably overlaps with that in the case of higher *p* (red lines in Fig. 1D). In summary, negative *S*–*E* relationships for the fractal RAD (Su, 2016) are significant. Decreasing *E* (faster or slower) with *S* are essentially affected by the fractal *p* (higher or lower) (lines in Figs. 1B, 1D, 1F and 1H).

Thirdly, rechecking empirical *S*–*E* relationships from the fractal angle (points in Figs. 1B, 1D, 1F and 1H) provide some additional information relative to the statistical analysis (Figs. 1A, 1C, 1E and 1G). *p* controls empirical *S*–*E* relationships for four indices (points in Figs. 1B, 1D, 1F and 1H), which is consist with *S*-*E* relationships for the fractal RAD (lines in Figs. 1B, 1D, 1F and 1H). Decreasing *E* (except *E*_Hill_) with *S* will be faster when *p* is higher (red points in Figs. 1B, 1F and 1H). Empirical *S*–*E*_Hill_ relationships cannot distinguish between the case of higher *p* and lower *p*, noting that red and blue points (higher and lower *p*) overlapped in Fig. 1D. Such forms are consistent with overlapping *S*–*E*_Hill_ relationships for the fractal RAD (please see red and blue lines in Fig. 1D).

In this study, empirical *S*–*E* relationships for four indices in three taxonomic categories are not consistent (Figs. 1A, 1C, 1E, 1G and Table 1), and their theoretical relationships for the fractal RAD are negative (lines in Figs. 1B, 1D, 1F and 1H). The conflict of *S*–*E* relationships between theory and observation is obvious. Simple linear relationships between *S* and four evenness indices vary in strength and magnitude across taxonomic categories (Table 1). The decreasing amplitude of *E* with *S* was controlled by the fractal *p*, and their relationships are unique for a given *p* (lines and points in Figs. 1B, 1D, 1F and 1H).

## Discussion

Understanding the causes of variation in diversity is one of fundamental aims of ecological research (Symonds & Johnson, 2008). Unfortunately, diversity is not as simple as might be expected (Smith & Wilson, 1996). Traditionally, species diversity was thought to consist of richness (*S*) and evenness (*E*) (Tuomisto, 2012). To make sense, *E* must be independent from *S* (Smith & Wilson, 1996). Thus, the *S*–*E* relationship had already attracted widely attention (Smith & Wilson, 1996; Stirling & Wilsey, 2001; Ma, 2005; Gosselin, 2006; Jost, 2010; Tuomisto, 2012; Zhang et al., 2012). However, there was surprisingly little consensus on this point (Stirling & Wilsey, 2001; Ma, 2005; Jost, 2010; Tuomisto, 2012).

It was not very clear which factor determined the *S*–*E* relationship. On one hand, measuring *E* was problematic (Symonds & Johnson, 2008). There were numerous methods by which *E* could be estimated according to different definitions of “evenness” (Smith & Wilson, 1996; Jost, 2010; Tuomisto, 2012). On the other hand, the *S*–*E* relationship could be affected by too many factors, such as taxonomic categories, spatial scale, migration rate, competition, predation, local species interactions, succession and so on (Stirling & Wilsey, 2001; Wilsey et al., 2005; Wilsey & Stirling, 2007; Zhang et al., 2012).

In this study, empirical *S*–*E* relationships for four different indices were not consistent among three taxonomic categories (Table 1), which were in line with many investigations (Stirling & Wilsey, 2001; Wilsey & Stirling, 2007; Zhang et al., 2012). Thus, taxonomic categories and different indices of *E* seemed not to be the key that determined the *S*–*E* relationship, although the strength and magnitude of their relationships varied with how the evenness was quantified and which taxonomic category was examined (Stirling & Wilsey, 2001; Wilsey & Stirling, 2007; Symonds & Johnson, 2008).

Investigating other factors (e.g., migration rate, local species interactions, competition and so on) would undoubtedly help to elucidate the determinants of the *S*–*E* relationship (Stirling & Wilsey, 2001; Wilsey & Stirling, 2007; Zhang et al., 2012). Unfortunately, there is no consensus (Ma, 2005; Symonds & Johnson, 2008; Jost, 2010; Tuomisto, 2012; Zhang et al., 2012). In fact, the lack of agreement on the determinants of the *S*–*E* relationship had been suggested as a limitation to understand the conflict between theory and observation (Stirling & Wilsey, 2001; Wilsey & Stirling, 2007; Jost, 2010; Tuomisto, 2012; Zhang et al., 2012). This lacuna also implied that the partitioning of diversity into *S* and *E* might not be settled without including additional factors (Stirling & Wilsey, 2001; Zhang et al., 2012).

The relative abundance distribution (RAD) is a basic feature of diversity (May, 1975; Magurran, 1988; Smith & Wilson, 1996). Smith & Wilson (1996) stated that *E* was the simplest method by which RAD could be measured. Some RAD models provided one of the most intuitively appealing measures of *E* (Peet, 1974). In other words, if RAD indeed determined *E* (Peet, 1974; Smith & Wilson, 1996; Gosselin, 2006), it could also be the key factor of the *S*–*E* relationship.

In this study, *S*–*E* relationships for the fractal RAD clarified that if the community had a fractal distribution of rank abundance, *E* would decrease with *S* (lines in Figs. 1B, 1D, 1F and 1H). This is consistent with *S*–*E* relationships for various RADs (Magurran, 1988; Gosselin, 2006) and many empirical investigations (Wilsey et al., 2005; Estrada-Villegas, McGill & Kalko, 2012; Zhang et al., 2012), which found that *S* and *E* were negatively related. More importantly, *S*–*E* relationships for the fractal RAD suggested that the decreasing amplitude of *E* with *S* was controlled by *p* (lines in Figs. 1B, 1D, 1F and 1H).

Rechecking empirical *S*–*E* relationships from the fractal RAD angle (points in Figs. 1B, 1D, 1F and 1H) originally indicated that empirically estimated *p* also had a great impact, which could be the most difference between previous studies and this one (Stirling & Wilsey, 2001; Wilsey et al., 2005; Gosselin, 2006; Wilsey & Stirling, 2007; Zhang et al., 2012). As noted before, *E* (except *E*_Hill_) declines faster with *S* when *p* is larger (red lines in Figs. 1B, 1F and 1H). The empirical *S*–*E*_Hill_ relationship also matched the overlapping pattern of their theoretical relationships for fractal RAD (Fig. 1D).

In brief, such results suggested that the fractal *p* controlled the *S*–*E* relationship from both theoretical and empirical perspectives (lines and points in Figs. 1B, 1D, 1F and 1H). Generally, the *S*–*E* relationship mainly depended on the pattern of RAD (Magurran, 1988; Gosselin, 2006). The *S*–*E* relationship would be unique for a given distribution, such as a particular model of RAD (e.g., the broken stick model), or a particular parameter of the RAD model (e.g., a specific *p*).

Since the pattern of RAD determined the *S*–*E* relationship, how to understand the conflict between theory and observation? How to explain the inconsistent *S*–*E* relationship in previous statistical analyses according to the negative *S*–*E* relationship in theory (lines in Figs. 1B, 1D, 1F and 1H)? The theoretical expectation of this paper was that the *S*–*E* relationship should be unique for a given RAD (Magurran, 1988; Gosselin, 2006). However, when different communities that matched different theoretical RADs (e.g., the geometric, the log series, and the random fraction distribution) or different parameters of the RAD model (e.g., different *p*) were put into one dataset, which form should be the *S*–*E* relationship for such dataset based on the ordinary linear regression?

Taking empirical *S*–*E* relationships in this paper (points in Figs. 1B, 1F and 1H) as an example, *E* (except *E*_Hill_) declines faster with *S* when *p* is larger (red lines in Figs. 1B, 1F and 1H). However, when the dataset consisted of some samples that matched different *p* (red and blue points in Figs. 1B, 1F and 1H), the *S*–*E* relationship would be inconsistent. The ordinary linear regression indicated that it could be positive (the *S*–*E*_Pielou_ relationship, Fig. 1A), negative (the *S*–*E*_var_ relationship of birds, Table 1) or uncorrelated (the *S*–*E*_Bulla_ and *S*–*E*_var_ relationships, Figs. 1F and 1H). Thus, the discrepancy between inconsistent *S*–*E* relationships in previous statistical analyses (Stirling & Wilsey, 2001; Ma, 2005; Wilsey & Stirling, 2007; Symonds & Johnson, 2008; Zhang et al., 2012) and their negative relationships in theory (Magurran, 1988; Gosselin, 2006) seemed to result chiefly from the dataset that matched different pattern of RADs. Inconsistent *S*–*E* relationships could be due to the variation of RAD in nature (May, 1975; Magurran, 1988; Tokeshi, 1993; McGill et al., 2007; Baldridge et al., 2016).

Finally, is the diversity a compound quantity made up of *S* and *E* (Smith & Wilson, 1996; Stirling & Wilsey, 2001; Jost, 2010; Tuomisto, 2012)? Since the pattern of RAD determined the *S*–*E* relationship, the decomposition of diversity into *S* and *E* would come down to whether or not there was a consistent general pattern of RAD across different taxonomic categories and environmental constraints. If this pattern existed, *S* and *E* should be correlated as the *S*–*E* relationship was unique for a given RAD (Magurran, 1988; Gosselin, 2006). In this case, the diversity could not be split into *S* and *E*. On the contrary, if this pattern really did not exist, the diversity could be split because the *S*–*E* relationship would be inconsistent when the dataset matched the different patterns of RAD.

Unfortunately, the performance of both sides was not very clear. On one hand, it was difficult to draw general conclusions about which model provided the best empirical fit to RADs (Baldridge et al., 2016). Over 40 models of RAD had been proposed on different theoretical grounds, and were all observed in real situations (May, 1975; Magurran, 1988; Tokeshi, 1993; McGill et al., 2007; Baldridge et al., 2016). Thus, the general pattern of RAD was unknown, because it was still an unsettled issue that which RAD model was the best (McGill et al., 2007; Baldridge et al., 2016). On the other hand, past attempts to fit RADs to empirical community data did not make the conclusion that that parameters of RAD models were always variable over communities. Actually, empirical studies on RADs established that the existence of a few common species and many rare species were very general (Magurran, 1988; Tokeshi, 1993; McGill et al., 2007; Baldridge et al., 2016; Su, 2016). Thus, it was unreasonable to expect that the general pattern must not exist (Magurran, 1988; Tokeshi, 1993; McGill et al., 2007; Baldridge et al., 2016; Su, 2016).

This study suggested that inconsistent *S*–*E* relationships were caused by the variation of RAD (Magurran, 1988; Gosselin, 2006; McGill et al., 2007). The partitioning of diversity into *S* and *E* should depend on the evidence that patterns of RAD were always various and a consistent general pattern must not exist. However, the current situation is that a consistent general pattern of RAD cannot be totally denied (Tokeshi, 1993; McGill et al., 2007; Baldridge et al., 2016; Su, 2016). Thus, it could be too early to split the diversity into *S* and *E* only based on the *S*–*E* relationship in statistical analyses. A more promising way to draw inferences about such controversial issue is likely to evaluate the performance of RAD models and increase the understanding of mechanisms that lead to the pattern of RAD (May, 1975; Magurran, 1988; Tokeshi, 1993; McGill et al., 2007; Baldridge et al., 2016).

## Conclusions

Magurran (1988) commented that “By looking at the fully species abundance distribution it is possible to get a better picture of the relationship between species richness (*S*) and evenness (*E*)”. Although various types of such relationships have been proposed (May, 1975; Magurran, 1988; Gosselin, 2006), this paper differed from previous studies in three ways. (1) The *S*–*E* relationship for the fractal RAD (Su, 2016) was firstly reported; (2) *S*–*E* relationships for various RADs were studied with simulated data (Smith & Wilson, 1996; Gosselin, 2006; Jost, 2010). This study assessed such relationships by using the raw data; (3) Empirical *S*–*E* relationships (Figs. 1A, 1C, 1E and 1G) were rechecked by the single parameter in the fractal model of RAD (in Figs. 1B, 1D, 1F and 1H).

This study suggested that *S*–*E* relationships were determined by the pattern of RAD (Magurran, 1988; Gosselin, 2006). Inconsistent *S*–*E* relationships were caused by the variation of RAD (Tokeshi, 1993; Baldridge et al., 2016). Since the general RAD is unknown, the decomposition of diversity into *S* and *E* is still open to doubt.

##  Supplemental Information

10.7717/peerj.4951/supp-1 Supplemental Information 1 The relationships between species richness (*S*) and four evenness indices (*E*_Pielou_, *E*_Hill_, *E*_Bulla_ and *E*_var_) in birds (Haila, 1983) indicated that empirical *S*–*E* relationships (left figures) were negative However, reevaluating their relationships from the fractal angle suggested that they were determined by the fractal *p*, which were consist with the *S*–*E* relationship for the fractal RAD. Four *S*–*E* lines (*S*–*E*_Pielou_, *S*–*E*_Hill_, *S*–*E*_Bulla_ and *S*–*E*_var_) according to the simulated data were shown in right figures when *p* = 0.6 (the bluest lines), 1.2, 1.8 and 2.4 (the reddest lines). *p* is the fractal parameter. Lower p means a slower decrease in *A*_*r*_ (the abundance of *r*-th species) compared with *A*_1_ (the abundance of dominant), and higher *p* indicates a rapid decrease, where *r* is the rank of species sorted down by species abundance.

10.7717/peerj.4951/supp-2 Supplemental Information 2 The relationships between species richness (*S*) and four evenness indices (*E*_Pielou_, *E*_Hill_, *E*_Bulla_ and *E*_var_) in fishes (Harrel, Davis & Dorris, 1967) indicated that empirical *S*–*E* relationships (left figures) were negative (*S*–*E*_Hill_ and *S*–*E*_var_ relationships) or unrelated (*S*–*E*_Pielou_ and *S*–*E*_Bulla_ relationships) However, reevaluating their relationships from the fractal angle suggested that they were determined by the fractal *p*, which were consist with the *S*–*E* relationship for the fractal RAD. Four *S*–*E* lines (*S*–*E*_Pielou_, *S*–*E*_Hill_, *S*–*E*_Bulla_ and *S*–*E*_var_) according to the simulated data were shown in right figures when *p* = 0.6 (the bluest lines), 1.2, 1.8 and 2.4 (the reddest lines). *p* is the fractal parameter. Lower *p* means a slower decrease in *A*_*r*_ (the abundance of *r*-th species) compared with *A*_1_ (the abundance of dominant), and higher *p* indicates a rapid decrease, where *r* is the rank of species sorted down by species abundance.

10.7717/peerj.4951/supp-3 Supplemental Information 3 The relationships between species richness (*S*) and four evenness indices (*E*_Pielou_, *E*_Hill_, *E*_Bulla_ and *E*_var_) in zooplankton (Akifumi Ohtaka & Shoichi, 1996) indicated that empirical *S*–*E* relationships (left figures) were negative (the *S*–*E*_Hill_ relationship) or positive (*S*–*E*_Pielou_, *S*–*E*_Bulla_, and *S*–*E*_var_ relationships) However, reevaluating their relationships from the fractal angle suggested that they were determined by the fractal *p*, which were consist with the *S*–*E* relationship for the fractal RAD. Four *S*–*E* lines (*S*–*E*_Pielou_, *S*–*E*_Hill_, *S*–*E*_Bulla_ and *S*–*E*_var_) according to the simulated data were shown in right figures when *p* = 0.6 (the bluest lines), 1.2, 1.8 and 2.4 (the reddest lines). *p* is the fractal parameter. Lower *p* means a slower decrease in *A*_*r*_ (the abundance of *r*-th species) compared with *A*_1_ (the abundance of dominant), and higher *p* indicates a rapid decrease, where *r* is the rank of species sorted down by species abundance.

10.7717/peerj.4951/supp-4 Supplemental Information 4 The computer code used to generate the figure

10.7717/peerj.4951/supp-5 Supplemental Information 5 Supplementary material Raw data from three sources.

10.7717/peerj.4951/supp-6 Supplemental Information 6 Statistical bird data The statistical bird data used to conduct four *S*–*E* relationships.

10.7717/peerj.4951/supp-7 Supplemental Information 7 Statistical fish data The statistical fish data used to conduct four *S*–*E* relationships.

10.7717/peerj.4951/supp-8 Supplemental Information 8 Statistical zooplankton data The statistical zooplankton data used to conduct four *S*–*E* relationships.

10.7717/peerj.4951/supp-9 Supplemental Information 9 Simulated data when *p* = 0.6 The simulated data used to conduct four *S*–*E* relationships (*S*–*E*_Pielou_, *S*–*E*_Hill_, *S*–*E*_Bulla_ and *S*–*E*_var_) when *p* = 0.6

10.7717/peerj.4951/supp-10 Supplemental Information 10 Simulated data when *p* = 1.2 The simulated data used to conduct four *S*–*E* relationships (*S*–*E*_Pielou_, *S*–*E*_Hill_, *S*–*E*_Bulla_ and *S*–*E*_var_) when *p* = 1.2

10.7717/peerj.4951/supp-11 Supplemental Information 11 Simulated data when *p* = 1.8 The simulated data used to conduct four *S*–*E* relationships (*S*–*E*_Pielou_, *S*–*E*_Hill_, *S*–*E*_Bulla_ and *S*–*E*_var_) when *p* = 1.8

10.7717/peerj.4951/supp-12 Supplemental Information 12 Simulated data when *p* = 2.4 The simulated data used to conduct four *S*–*E* relationships (*S*–*E*_Pielou_, *S*–*E*_Hill_, *S*–*E*_Bulla_ and *S*–*E*_var_) when *p* = 2.4

10.7717/peerj.4951/supp-13 Supplemental Information 13 The computer code used to estimate the fractal *p*

## Additional Information and Declarations

### Data Availability

The following information was supplied regarding data availability: The raw data are provided as Supplemental Files.
