# Supplementary material for: A relationship between species richness and evenness that depends on specific relative abundance distribution
Source: PeerJ. 2018 Jun 12;6:e4951. doi: 10.7717/peerj.4951 (PMC6003394; doi:10.7717/peerj.4951)
Supplement: Supplemental Information 3 — However, reevaluating their relationships from the fractal angle suggested that they were determined by the fractal p, which were consist with the S–E relationship for the fractal RAD. Four S–E lines (S–EPielou, S–EHill, S–EBulla and S–Evar) according to the simulated data were shown in right figures when p = 0.6 (the bluest lines), 1.2, 1.8 and 2.4 (the reddest lines). p is the fractal parameter. Lower p means a slower decrease in Ar (the abundance of r-th species) compared with A1 (the abundance of dominant), and higher p indicates a rapid decrease, where r is the rank of species sorted down by species abundance. [file peerj-06-4951-s003.pdf]

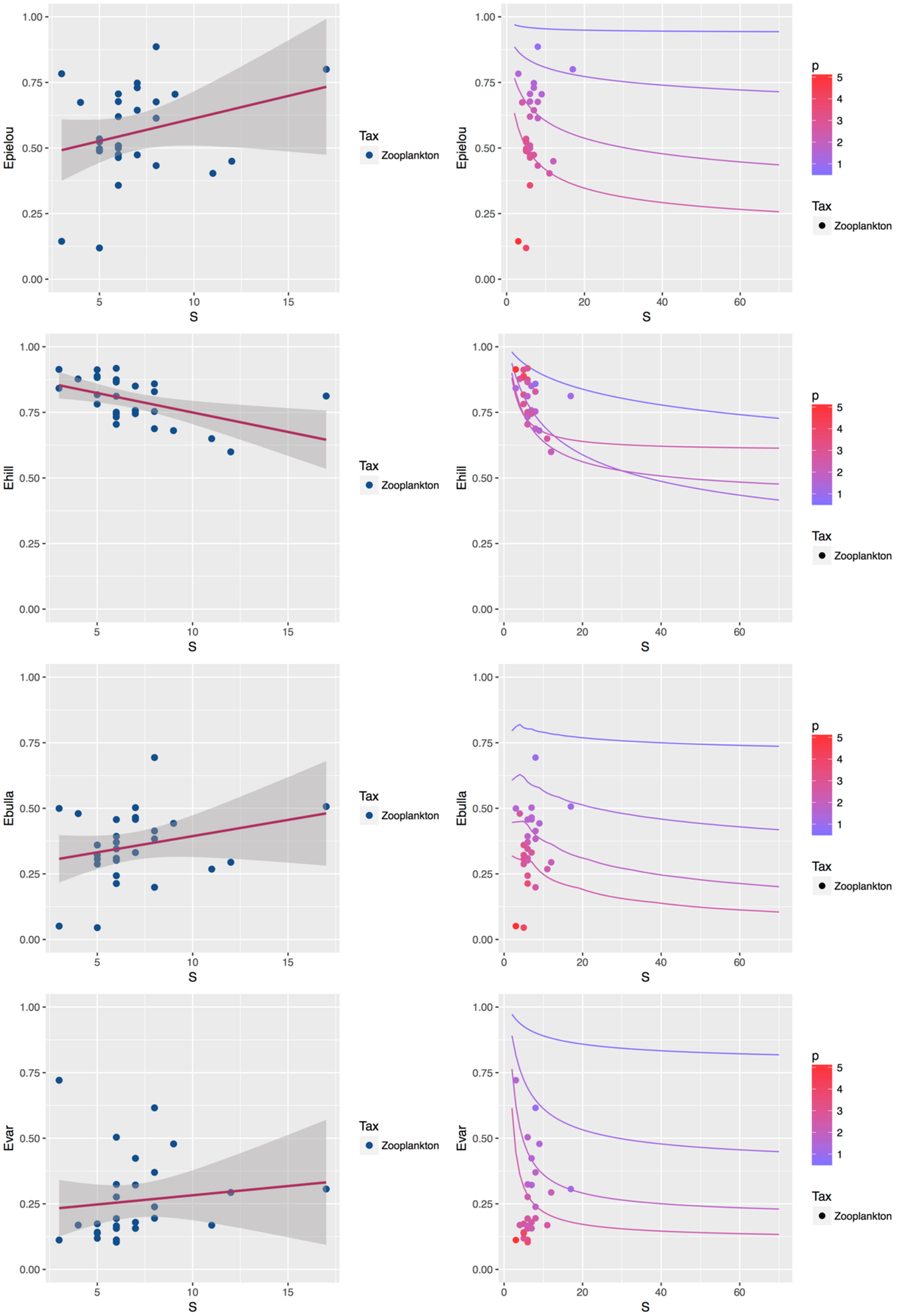
Epielou
Ehill
Ebulla
Evar
S
Tax
Zooplankton
p
1.00
0.75
0.50
0.25
0.00
5
10
15
0
20
40
60
1
2
3
4
5
